# Improving Diagnosis Through Digital Pathology: Proof-of-Concept Implementation Using Smart Contracts and Decentralized File Storage

**DOI:** 10.2196/34207

**Published:** 2022-03-28

**Authors:** Hemang Subramanian, Susmitha Subramanian

**Affiliations:** 1 Department of Information Systems and Business Analytics Florida International University Miami, FL United States; 2 Department of Clinical Pathology Apollo Super Speciality Hospitals Bangalore India

**Keywords:** digital pathology, nonfungible token standard, decentralized storage, security and patient data confidentiality using design, pathology, storage, security, confidentiality, data, design, diagnosis, proof of concept, implementation, software, blockchain, limitation, privacy

## Abstract

**Background:**

Recent advancements in digital pathology resulting from advances in imaging and digitization have increased the convenience and usability of pathology for disease diagnosis, especially in oncology, urology, and gastroenteric diagnosis. However, despite the possibilities to include low-cost diagnosis and viable telemedicine, digital pathology is not yet accessible owing to expensive storage, data security requirements, and network bandwidth limitations to transfer high-resolution images and associated data. The increase in storage, transmission, and security complexity concerning data collection and diagnosis makes it even more challenging to use artificial intelligence algorithms for machine-assisted disease diagnosis. We designed and prototyped a digital pathology system that uses blockchain-based smart contracts using the nonfungible token (NFT) standard and the Interplanetary File System for data storage. Our design remediates shortcomings in the existing digital pathology systems infrastructure, which is centralized. The proposed design is extendable to other fields of medicine that require high-fidelity image and data storage. Our solution is implemented in data systems that can improve access quality of care and reduce the cost of access to specialized pathological diagnosis, reducing cycle times for diagnosis.

**Objective:**

The main objectives of this study are to highlight the issues in digital pathology and suggest that a software architecture–based blockchain and the Interplanetary File System create a low-cost data storage and transmission technology.

**Methods:**

We used the design science research method consisting of 6 stages to inform our design overall. We innovated over existing public-private designs for blockchains but using a 2-layered approach that separates actual file storage from metadata and data persistence.

**Results:**

Here, we identified key challenges to adopting digital pathology, including challenges concerning long-term storage and the transmission of information. Next, using accepted frameworks in NFT-based intelligent contracts and recent innovations in distributed secure storage, we proposed a decentralized, secure, and privacy-preserving digital pathology system. Our design and prototype implementation using Solidity, web3.js, Ethereum, and node.js helped us address several challenges facing digital pathology. We demonstrated how our solution, which combines NFT smart contract standard with persistent decentralized file storage, solves most of the challenges of digital pathology and sets the stage for reducing costs and improving patient care and speed of diagnosis.

**Conclusions:**

We identified technical limitations that increase costs and reduce the mass adoption of digital pathology. We presented several design innovations using NFT decentralized storage standards to prototype a system. We also presented the implementation details of a unique security architecture for a digital pathology system. We illustrated how this design can overcome privacy, security, network-based storage, and data transmission limitations. We illustrated how improving these factors sets the stage for improving data quality and standardized application of machine learning and artificial intelligence to such data.

## Introduction

### Background

Digital pathology, a subfield of pathology, deals with information management enabled by digital technologies such as high-resolution imaging, computer storage, and network connectivity [1,2]. In digital pathology, the information generated from digitizing slides containing microscopic images of biological samples is shared over a computer network, with the expectation of faster diagnosis and remote access to medical experts. Digital pathology has several advantages over conventional pathology, which bases diagnosis on glass slides and microscopic examination of images on slides [3]. In digital pathology, extremely high-resolution images enable physicians to tag, expand, share, and analyze specific sections of the image slides to diagnose a disease. More recently, the deployment of deep learning algorithms to recognize patterns in high-resolution digital images has been shown to reduce the time taken to diagnose particular diseases [3,4]. Digital pathology also allows pathologists and referring physicians to share data with other experts in clinical diagnostics to concur concerning diagnosis and treatment pathways for specific diseases. As an example, trained superspecialists in oncological pathology, such as the coauthor (SS) of this paper, can provide a very detailed clinical diagnosis of the specific type of cancer, the rate of spread, the speed of spread, the stage, and the types of cells and tissues affected by detailed examination of slides.

Furthermore, the slides and diagnosis can be shared with other senior experts to confirm the diagnosis. These specialists can further suggest and approve treatments for the patients, thus reducing cycle time and improving diagnosis accuracy through telediagnosis [5]. Thus, digital pathology can provide an accurate, timely, and concise diagnosis of the disease while providing the ability to share such diagnoses with other specialists.

**Figure 1:**
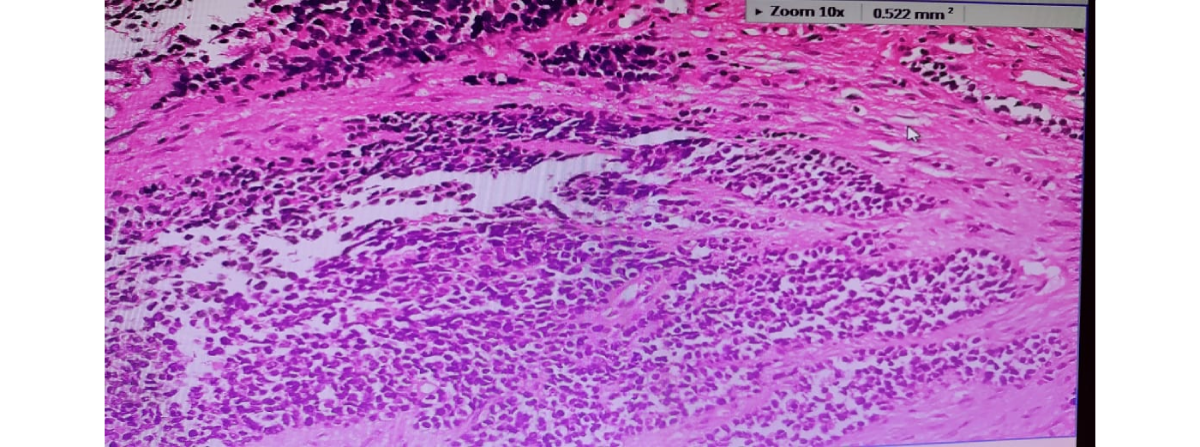
An illustration of a digital pathology image from a slide used in an actual diagnosis by the coauthor (SS). The image is from a high-resolution microscopic scan and digitized slide that is presented to the clinical pathologist on a computer screen for further diagnosis.

Digital pathology uses technology that includes high-resolution remote and internet-based microscopy and high-resolution scans of glass slides, which can have multiple advantages. First, digital pathology benefits patients in locations where access to medical care is challenging and where the number of clinicians, physicians, qualified pathologists, and hospital systems are scarce [6]. Second, digitizing images at high resolution provides physicians and specialists with ease of use, thereby reducing the use of a microscope. However, they can view high-resolution images on a computer screen, thus reducing fatigue to the eye [1,7]. Third, physicians and doctors can focus on specific areas of the digital image by using input-output devices such as keyboards, mice, and digital pencils to write notes on specific areas of the images. Figure 1 shows the images and magnified sections of imaged slides that pathologists use to diagnose the medical condition. Fourth, physicians can share such data with other experts or senior practitioners in the field, who can later arrive at a consensus concerning the diagnosis about the progress of disease and recommend treatment pathways for patients. Owing to these advantages of personal ease of use and shareability, the role of digital pathology in providing timely, accurate, and concise prognosis and pathways to clinical recovery has been recognized by the medical fraternity. Hospital systems are slowly transitioning to a fully digital diagnosis mode, reducing physical storage requirements for glass slides and partially automating the transmission, attainment of agreement among experts, and treatment pathway recommendations for patients [5].

### Infrastructure Challenges for Digital Pathology

However, there are several challenges with the adoption of digital pathology to make it widely accessible. These challenges relate to the transmission, storage, retrieval, and uniform clinical diagnosis using algorithms from slide or slides and image or images derived from tissue and biosample scans. Digital image scans are made possible by remote and internet-based microscopy or through a high-resolution scan of microscopic slides into images of much higher resolution (100,000 dpi to 1,000,000 dpi). Such scanned images also require high-resolution storage, for the data not to be compressed. While transmitting such high-resolution images, transmission could lead to data loss if images are compressed and decompressed, potentially affecting the consensus for diagnosis across the network. Compression and decompression of data lead to loss of information, which can potentially lead to an inaccurate diagnosis or inaccurate data training from these samples [8]. In addition, inaccurate images and matched diagnoses create flawed training data sets, reducing the prediction accuracy when deep learning models are applied to such data sets. Although network-attached storage, cloud-based storage, commercial databases, and workflow systems such as enterprise resource planning frameworks are plausible solutions to accomplish security and transmissibility of related data (ie, image scans or diagnoses), these centralized software systems are expensive to own and increase the total cost of ownership for pathological systems, which makes disease diagnoses via pathology unaffordable [6].

In centralized systems, the cloud provider or database network administrator controls the security of the network and patient data. Such centralized data control could often violate patient privacy and be susceptible to security breaches, ransomware attacks, and other data security problems. As a result, both the storage and network-based transmission of such high-resolution images are challenging. Second, obtaining the necessary audit or data transmission logs of such algorithms is difficult in centralized systems, which are database-centric and often need to interface between different hospital and clinical systems managed by different entities [9,10]. Third, although most research in the digital pathology area has automated deep learning techniques for pattern recognition with images, such application of algorithms will only fructify when a sufficient corpus of images combined with human-expert classification or training is present. Specialized diagnosis required to detect rare diseases such as sickle cell disease or certain types of cancer such as bone marrow cancer makes it increasingly challenging to obtain large training samples of digital images in high-resolution biosamples with human classification. A decentralized, secure, and standardized storage of high-resolution images and appropriate expert classification of such data that includes specific sections of tissue scans, physically marked and noted with the clinical disease diagnosis, can potentially solve this problem. Such uniform storage of images, diagnoses, and associated metadata information such as sections of the images, which help physicians diagnose the disease, is essential. Such data can lead to the creation of a proper training data set for deep learning, which can enhance the capabilities of automated disease diagnosis in the future. The fields of artificial intelligence and machine learning, such as deep learning, are applied to remote and internet-based microscopy–derived images, using high-quality training samples, wherein the trained models can aid in faster diagnosis [1,3,4,11]. Such machine-assisted diagnoses can aid physicians by complementing their knowledge through pattern detection.

This paper highlights the challenges in digital pathology associated with secure data storage, secure data transmission, data privacy, and storage and transmission of high-resolution images and associated diagnosis from physicians. Furthermore, we propose a unique design with smart contracts and the Ethereum blockchain, which uses the Ethereum Request for Comments (ERC)-721 standard to store and transmit images while tracking ownership [12]. We propose an innovative decentralized storage and encryption scheme separate from the public blockchain [13]. We also document how these challenges can be overcome. We outline why and how such a design using smart contracts and blockchains can overcome issues in training accuracy using machine learning and artificial intelligence techniques for pattern detection from slides with a higher image resolution.

### Advantages of Digital Pathology

The primary responsibility of practicing pathologists is to maintain the confidentiality of patient data and diagnoses. In digital pathology, it is a typical pattern that multiple pathologists and diagnostic programs have access to the data uploaded from one of the following sources: (1) high-resolution tissue photography, (2) scanned slides that constitute images from microscopes, or (3) remote and internet-based microscopes that image human tissue samples and store and transmit these images. The patient’s medical record and diagnosis should be shared with those with the *right to know*. Data stored are expected to adhere to laws such as Health Insurance Portability and Accountability Act and General Data Protection Regulation. Similarly, only the pathologist who accesses the digital pathology platform should view these high-resolution images, make modifications, and make a diagnosis. Although pathologists and other specialists can access these images and slides on a case-by-case basis, the primary owner of the data is the patient. The patient can choose to remove access to the physician, or the hospital system might invalidate the data access after a legally mandated time frame (eg, 3 years).

**Figure 2:**
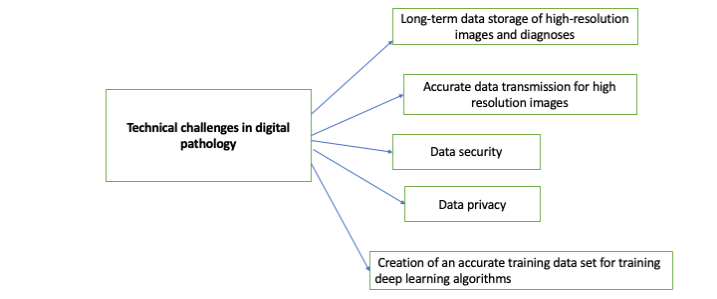
Technical challenges in digital pathology.

In addition, these images should be stored only for an extended period, as mandated by law, and retrieved cost-effectively for purposes such as reference [5]. As discussed in the previous section, these high-resolution images will require significant storage and secure high-bandwidth transmission to transfer images, diagnoses, and audit logs. If these conditions are not met, digital pathology costs will become financially unviable to adopt and use for diagnosis. In the long term, storage requirements and cost reduction are essential for pathologists to ensure data accuracy, privacy, and access control [9,10]. Long-term storage costs, the transmission of high-resolution images about digital pathology to different owners on the network, and the high costs associated with the security, privacy, and auditability of such data create 3 main issues for the success of digital pathology as an effective practice. The challenges in digital pathology are summarized in Figure 2.

In digital pathology, the image resolution of slides containing images of tissues or other human body samples such as blood vessels is comparatively high. It often ranges between 1× and 400× of slides obtained using traditional microscopy in nondigital pathology [14]. The advantage of such platforms is that multiple pathologists can simultaneously access the slide if they have the corresponding software and authorized access to the slides. In addition, pathologists can share independent diagnoses on the web after analyzing these images. As a result, significant time is saved in prognosis or diagnosis and the treatment compared with scenarios where microscopic glass slides would have to be transported physically. A second or third opinion must be obtained to draw a fail-safe conclusion if the severity of the disease is high, in which case reports, images, and other patient data must be shared by maintaining the privacy and patient confidentiality protocols.

Artificial intelligence algorithms can aid physicians in detecting patterns from images, especially in pathology, where cancer growth creates patterns in body tissues. Deep learning models can be trained with sufficient samples to detect and code sections of images with a specific diagnosis [3,11]. Such models can recognize patterns of spread, the spread stage, and the spread level in cancerous cells after appropriate training from a large corpus of samples that physicians have accurately classified. In addition, prognostic factors that can aid in treatment can be determined. Although storing and documenting preliminary case data for human diagnosis is equally important, such data can later be used downstream as inputs to training and classification algorithms to automate the detection and diagnosis of pathological conditions. The use of deep learning algorithms such as convolutional neural networks and recurrent neural networks combined with support from graphical processing units offered by NVIDIA and AMD provides significant strength to such automated analysis [3,11]. Often, such trained algorithms have lower accuracy than required because of the uniqueness of individual cases. If irregular patterns do not fit into the diagnosis, then such cases have to be manually studied, and such cases could be labeled independently as a rare possibility.

**Figure 3:**
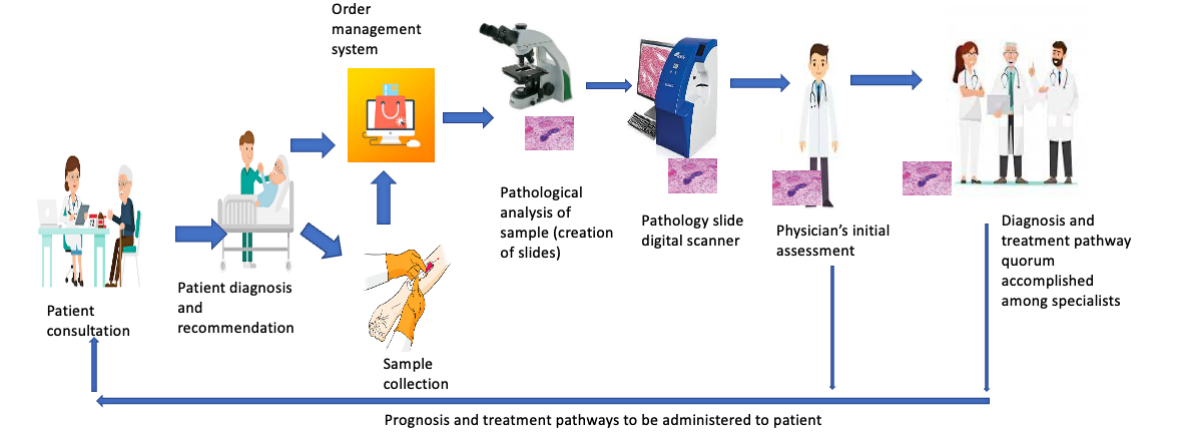
Workflow of digital pathology system.

The goal of such a digital pathology could evolve to enable automatic diagnosis through the accrual of a proper training data set and human classification algorithms. When such data sets become available and deep learning algorithms are trained appropriately on human-classified data, the time to diagnosis will naturally decrease. In addition, data from digital pathology can be used for multiple purposes such as teaching, research, and continuing medical education seminars. Here, data retrieval is fast; access is provided on a distributed platform that caters to both privacy and security, and search and retrieval and display of data in the digital formats used in traditional pathology is proper. Figure 3 shows a sequence diagram that captures a typical pathology workflow for diagnosing a patient’s condition using digital pathology.

Pathologists can choose to expand and focus on certain sections of the slides at a higher resolution to diagnose the disease involved and create notes on these slides by annotating sections of the scanned images. Later, multiple pathologists or other specialists can view the same slides to create a diagnosis and discuss treatment pathways. These modified slides with annotations from experts are stored back in the centralized system for retrieval and use, in other cases, for research or teaching purposes.

### Blockchains, Nonfungible Tokens, and Decentralized Storage (Interplanetary File System) Applied to Health Care

Blockchains address the challenges of system interoperability, security, privacy, nonrepudiation, storage persistence, decentralized storage, decentralized validation of transactions, and accessibility for all data [13]. Before the advent of blockchains, anonymized and privacy-controlled single point of access for multiple data sources was provided for each user. Similarly, assuring privacy and user-controlled data and tracing or auditing data transmission records was difficult because the data resided in private centrally controlled repositories. Such data repositories are administered by a central administrator, who is often the single weak link for ensuring data security and privacy. In medical record storage systems, owing to multiple disparate administration end points involving different organizations, data are shared among multiple administrators, creating a weak link for a privacy violation. Because blockchains remove the need to centralize data storage and control data flow and modifications, they have several applications in health care [15]. Blockchains have been shown to provide a cost-effective and robust decentralized technology for a variety of health care applications, such as tracking clinical trials [16], enabling tamper resistance [17] for mobile health data, removing trust deficiencies during the COVID-19 pandemic [18], and eliminating substandard drugs from the supply chain [19].

Blockchains have evolved to support programming logic by including variables and loop structures using high-level programming languages such as Solidity [20] and a remote and internet-based machine that interprets and executes the compiled programs. These programs are compiled and deployed onto remote and internet-based machines that validate transactional and semantic logic in programs. Remote and internet-based machines understand opcodes (or machine-interpretable code), which are outputs of a compilation of a higher-level programming language such as Solidity. Examples of blockchains include Bitcoin (which supports a rudimentary scripting language), Ethereum, Cardano, and Solana. These smart contracts enable web- or mobile-based decentralized applications that interact with the underlying blockchain and remote and internet-based machine based on user input. Although blockchains such as Hyperledger, Ethereum, and Cardano have been shown to support data storage and programmability of contracts, it remains challenging to ensure that such data storage can happen cost-effectively on the blockchain (on-chain storage). The governance structures of public blockchains require application writers and data creators to pay a transaction fee, which is often proportional to the bandwidth and space used on the blockchain.

This is because storing even small amounts of binary data on a public or private blockchain, that is, on-chain, as a transaction record would cost several hundred dollars. Although several papers in the medical informatics field have chosen this costly approach to store data on the blockchain (or have used a Hyperledger framework with a private blockchain or a consortium blockchain to store data), such storage mechanisms are inefficient for digital pathology, which requires more extensive storage and other challenges, as shown in Figure 2.

This is because image storage requires large quantities of cost-effective data storage. Image transmission on the network with such large images is nearly impossible, given today’s email client and other networking protocols, such as Simple Mail Transfer Protocol. The only possible likelihood is to store images from a local repository that distributes the image onto a network file storage and makes the file addressable.

Another challenge with such an approach is that the data stored in the blockchain are public information and can be effectively downloaded from the remote and internet-based machine by any user as it is stored. Therefore, unless the stored data are encrypted in an unidentifiable way using the user identifiers of both the sender and the recipients, such data are of no use. In this study, we propose a 2-layer blockchain solution for nonfungible tokens using the ERC-721 standard for nonfungible tokens (NFTs) that separate metadata from file storage and promises authenticity, security, distributed storage, and ease of use [21]. We use this 2-level architecture that provides affordability for use cases such as digital pathology, which requires extremely high-resolution and high-density storage. In addition, we use an existing well-used standard supported by sizable open-source marketplaces in the context of digital art to support all use cases necessary to create a cost-effective digital pathology system. Such a system will provide traceability of each transaction by using transaction records and logs and cost-effectively enable storage and transmission. We propose application-level encryption of metadata URI’s stored on-chain and associated with the nonfungible token. We prototype a system and demonstrate how such a decentralized workflow for storage, retrieval, and transmission occurs while catering to various design requirements for such a system.

### Decentralized Data Storage Using the Interplanetary File System

Although blockchain technology provides several advantages noted above and health care can be shared efficiently among peers (hospitals or physicians) in a health care system, there has never been a justification to store such health care data on-chain [22]. Particularly in digital pathology, where high-resolution image scans require significant storage, the feasibility of storing data on-chain is impossible because of the infrastructure costs involved in storing, validating, and retrieving such data. Particularly for mass-adoption blockchains such as Ethereum, Solana, and ADA, data storage and transaction fees would significantly exceed the affordability of the digital service compared with the traditional mechanism of glass slides. A simple estimate for the Ethereum blockchain stated that it costs between US $17,000 and US $76,000 to store 1 MB of data on-chain. In addition, the blockchain does not provide version control or other features to optimize data storage such as the Interplanetary File System (IPFS). The operation of the IPFS for file storage and retrieval is described as follows.

When a file is added to the IPFS, it is split into smaller chunks, cryptographically hashed, and given a unique fingerprint called the content identifier (CID). The CID acts as a permanent record of the file as it currently exists. When other nodes (or a program using JavaScript Object Notation and Remote Procedure Call) look up the file using the unique CID, they ask their peer nodes about storing the content referenced by the file’s CID. The IPFS provides a mechanism to identify (pin) records to hosts on the network. If the content is not pinned, it can be deleted to access the more recently accessed files. Versioning of files is maintained by creating a new CID because of which files stored on the IPFS are resistant to tampering and censorship on the network. The IPFS uses a distributed hash table to identify addresses and images. The IPFS facilitates the removal of duplicate files and enables the creation of version-control history [2]. The IPFS optimizes the storage and retrieval of frequently accessed files through a cache mechanism, where frequently accessed files are prioritized using hash tables that rank files based on their access frequencies. On the IPFS, each file can be individually accessed with its hash address from the internet; the IPFS is a content-addressed block storage system with features such as high throughput, security with hash mapping of transactions, and concurrent access of files.

### NFT Standard for Digital Pathology

The NFT standard is a unique and noninterchangeable unit of data (or a data pointer) stored on the blockchain. Although other token standards such as the cryptocurrency token standard ERC-20 have been popular, the NFT standard authenticates and associates reproducible digital files such as photos, videos, and audio on the Ethereum blockchain. The ERC-20 standard is used to implement the cryptotoken functionality. It is usually used to create a token for sourcing funding during an initial coin offer and other such uses, enabling token holders to participate in blockchain governance. The major feature of ERC-20 is that it is tradable against any other ETH-compatible token, on crypto exchanges, or coin-swapping liquidity provider platforms such as Uniswap. As a result, we have to use the ERC-721 or NFT standard to implement the image storage, authentication, ownership, and digital rights in the context of digital pathology. NFTs use a digital ledger to provide a public certificate of authenticity or proof of ownership. The NFT standard has wide adoption for various multimedia-based token creation and has seen significant adoption among the art collectors. Similarly, more recent innovations in virtual reality, augmented reality, and the metaverse have enabled the creation and sale of digital assets using this standard.

Overall, if we store the characteristics of the image off-chain on the IPFS (eg, in a metadata file) and have the metadata’s unique CID referenced on the blockchain and then minted into an NFT, then it is possible to separate token ownership from token access. The ownership is documented in the metadata file, whereas the access is implemented via functions supported by the ERC-721 standard. This architecture provides us with the flexibility to enable the separation of ownership of the token from the access properties. It also enables us to securely transfer access (or replicate access) after signing the token by the owner, enables token burn, and assigns access to others. In addition, such a design enables the process of minting, replication, and burning of the token. The NFT standard uses these two technologies, that is, the NFT standard smart contract on the Ethereum blockchain and the IPFS for decentralized file storage.

### The Goal of This Study

In this paper, we analyze the following two research questions (RQs) using the design science research methodology:

RQ1: How does blockchain-enabled digital pathology improve data sanctity, privacy, data validity, and cost-effective, secure network storage?

RQ2: Does such a system design improve physician diagnosis by reducing the cost of storage, improving security diagnosis times, and improving machine learning capabilities by improving the accuracy of data stored?

## Methods

### Overview

**Figure 4:**
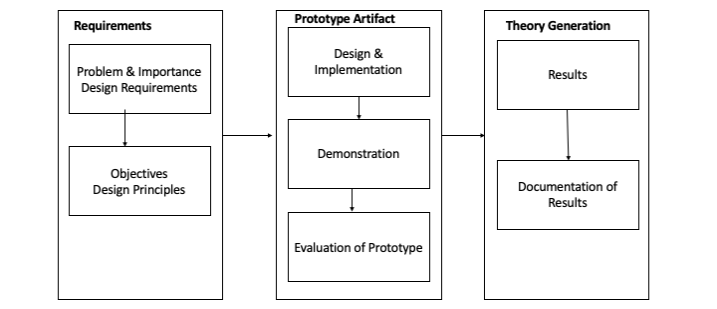
Diagram represents the steps in the design science research methodology.

We use the design science research process (DSRP) methodology used in information systems and computer science disciplines and expert recommendations. Our solution prototype proposes an innovative smart contract–based digital pathology system using public infrastructure to simplify high-resolution image storage and provide a decentralized, secure, and privacy-preserving data-sharing solution. Figure 4 shows the research methodology [23].

DSRP is a well-established research methodology in information systems, computer science, and information technology management. It is used mainly to create design-based system solutions for practical problems, for which there are no previous solutions. As the name states, DSRP applies design science to create a software- or system-based prototype that addresses the research problem. Using the prototype, researchers validate the assumptions they made. We use the following steps in DSRP to implement and test our prototype: First, we describe the problem and define its importance. The challenges in digital pathology are described in the *Introduction* section. We obtain the problem definition (challenges) from practicing pathologists—the coauthor (SS) of this paper—through a formal procedure of requirement gathering. We share a questionnaire about using the digital pathology system and record how they faced challenges in day-to-day work. Second, the objectives of the solution are clarified in terms of the benefits of improving the quality of care and maintaining accurate data for creating clean data sets. These questions are documented in Multimedia Appendix 1. Table 1 defines the key problems that we aim to address using our solution and the main objectives.

In step 3, we design and implement a proof-of-concept use principles of systems analysis and design to create a model-view-controller architecture–based system with JavaScript, web3.js, node.js, and Ethereum stack combined with the IPFS for long-term data storage. The following section describes the creation of the NFT-based IPFS that meets the various objectives of the solution. We later discuss the confidentiality requirements and application-level encryption of metadata files for storage. In the fourth step, we demonstrate the artifact and the solution prototype. Fifth, we evaluate the artifact for whether it indeed met all the design objectives for the problem it indeed set out to solve. Finally, we document the results of our experiments and describe how our solution benefits the wider digital pathology community and the contributions of this study. We also believe that our solution can be appropriated for other high-density storage requirements in the medical world.

**Table 1:** Problem definition and objectives.

Problem definition	Importance and business outcome	Objectives of the solution
Long-term distributed and cost-effective storage of high-resolution images, metadata, and diagnoses provided by multiple users	Reduce the cost of adoption and utilization	Evaluation of alternate technologies that provide such storage; possibly separate the storage from the data transmission mechanism
Network transmission or the ability to share such high-resolution images and associated data	Improve speed of diagnosis for the disease	Mechanism to share data with others through the internet in a secure way
Data security	Improve security for all clinical data and provide access control	Mechanism to prevent unauthorized access of data
Data privacy	Improve the privacy of data in the system such that only the physician, pathologist, and patient can access these data	Mechanism to share the data on a need-to-know basis
Ability to maintain audit data transmission or data from shared access logs	Improve compliance using Health Insurance Portability and Accountability Act, General Data Protection Regulation, and privacy laws	A mechanism to trace how data have been shared among different users; store of logs should be perennial
Ability to maintain accuracy for creating a high-quality training data set for future application to artificial intelligence or deep learning models	Better training data can be stored on the network, and such data can facilitate better machine learning and artificial intelligence prediction	The mechanism above should have the ability to be audited, verified, and analyzed by third parties independent of the system
Reduce cost to the patient	Public infrastructure with well-established cryptographic protocols can enable the solution to scale significantly; network transmission is avoided by design	Network-attached storage costs are reduced for the solution; telemedicine and remote diagnosis are enabled easily
Improve the quality of care for the patient	Once higher accuracy for training data is established, the quality of care for the patient would have improved significantly	Machine-assisted diagnosis is possible
Ability to separate artifact ownership from token access	The pathological data record should explicitly state that the patient is the data owner, regardless of other individuals accessing the record	The metadata always explicitly records the ownership of the digital image by storing the patient’s pseudonymous information such as ID or wallet address that are unique to the patient
Ability to remove access after a prespecified time interval as per the legal and regulatory conditions	After a prespecified interval, the record should automatically be burned for all nonowners; those health care professionals who received the record should no longer have access to it	This functionality provides the ability to remove access to specific records and adhere to health care data storage and retrieval laws

### Architecture and Design Components

**Figure 5:**
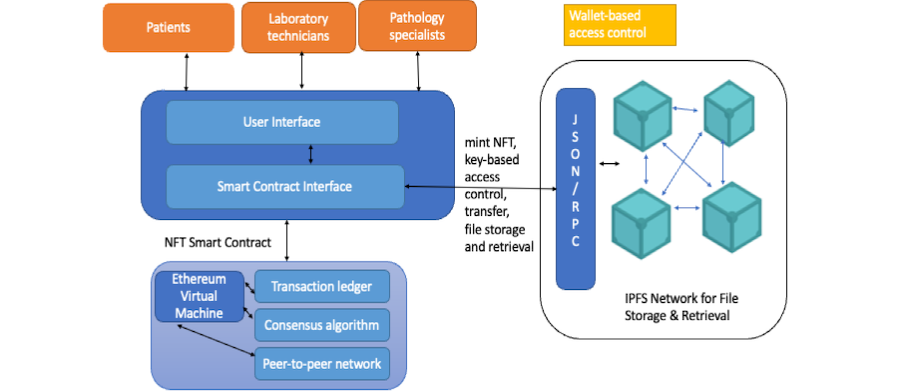
Software architecture of the digital pathology system with blockchain and IPFS used for tokenizing, storing, and sharing access to the digital outputs of the health care system. IPFS: Interplanetary File System; NFT: nonfungible token.

Figure 5 depicts the software architecture of the decentralized digital pathology system. The system architecture conforms to the model-view-controller architecture pattern, and the system’s main components are described in the next section.

### Description

We use a model-view-controller software architecture pattern [24] to prototype the digital pathology system capable of addressing the requirements described in the *Introduction* section. The model stores the data about image scans and uses the interfaces provided by the view and controllers. Data are stored on the IPFS [25] and are usually of two types:

1. High-resolution image files about scans and any modifications made by the pathologist or other professionals, such as notes and image markings, to identify specific disease patterns, and
2. The JSON files containing metadata about the scans stored on the web or the diagnosis provided by the physician.

The IPFS provides a decentralized store for data and is based on caching data where file access frequency provides users with performant file storage.

We use Solidity programming language for programming the smart contract, which runs on the Ethereum test network. We use several JavaScript toolkits, namely, web3.js, node.js, and essential libraries associated with HTML5 and bootstrap.css, to code the front end. For testing the prototype, we used the Ganache command line toolkit (CLI), the VSCode library extensions for Solidity and HTML5 and JavaScript, and the integrated development environment for programming using Remix smart contract editor. We use the Ethereum Kovan and Ropsten test networks to implement the prototype and to validate our solution against the objectives set out in this paper.

The user interface provides a visual interface for physicians, laboratory technicians, and patients to view the data that they own or share with their wallets. While physicians, laboratory technicians, and patients can access the same image addressed using the CID on the IPFS, the main controlling file is the JSON, which interacts with the smart contract infrastructure and mints the token. The address stored (ie, the CID of the JSON file that contains metadata about the image or diagnosis) in the minted token is hidden from public access by encrypting the CID with the user’s keys. The security aspects of the proposed system are described in the following section. The user interface then interacts with a controller consisting of a series of smart contract methods that mint new tokens, transfer tokens from the laboratory technician to the physician and from one physician to another using the NFT protocol, and so on. The decentralized application is responsible for creating newer images and minting the NFTs, which can potentially generate notes from physicians, clinical pathologists, or patients to enable data to be stored within the IPFS. This data model is described in the JSON file and indicates the different types of files used by such a system.

Because the IPFS stores each image file separately (as a unique addressable and unalterable hash), a JSON schema file similar to code listing 1 (Multimedia Appendix 2) creates a JSON file that contains the address for the CID for each NFT. Each time a new file is added, a new JSON metadata file is generated and stored within the IPFS. As a caveat, although the IPFS is a public infrastructure consisting of thousands of nodes from voluntary contributors, token-based decentralized storage mechanisms such as FileCoin and Storj are also available, providing decentralized storage and enabling application developers to access guaranteed distributed storage on the network. Both Storj and FileCoin incentivize users to participate in decentralized file storage by rewarding contributors with cryptocurrency. The price for Storj is approximately US $4 per month for 1-TB storage and US $7 per month per TB of bandwidth, which is lower than most other web hosting or cloud service provider costs, thereby reducing storage and network transmission costs for high-resolution images.

From code listing 1, that is, the JSON metadata file, we obtain a unique CID that addresses each image location along with the pseudoidentity of the owner of the image in the form of the owner’s name and wallet ID. This information is unique and used to separate ownership from access rights. The blockchain provides the encryption and security for this JSON metadata file of the images used in the data set.

The CID of the metadata file is used as the input to the NFT mint (ERC-721) and provides the originator of the file access to this token. This token can then be transferred to other individuals on the network by simply using their wallet addresses. Every JSON metadata file associated with the record should contain the owner name and ID, and the patient’s public wallet address that uniquely identifies the patient. When the metadata file must be shared among multiple people, the sender will have to mint multiple tokens with separate metadata files (CIDs) to transfer the token to other individuals. However, the base file will remain the same, and the image data file is referenced in each JSON file independently. The source code for minting the NFT in Solidity is listed in code listing 2 (Multimedia Appendix 2).

The original NFT can be transferred by sending the token to the recipient’s wallet address using the *SafeTransfer* function described in code listing 3 (Multimedia Appendix 2).

### Decentralized Application Functions to Mint, Store, Transfer Ownership of, and Expire (Burn) the Token

In the decentralized app, we use the smart contract code of the NFT standard (ERC-721) to mint an NFT on the Ethereum network and use the JSON file’s IPFS URL (code listing 4 in Multimedia Appendix 2). The web interface invokes the smart contract from the Ethereum remote and internet-based machine to transfer the token from the source address to the destination address. Further, we transfer the token ownership to a receiver’s blockchain wallet. This mechanism ensures that only the NFT owner has access to the JSON metadata file, which points to the IPFS URI of the pathological image sample. Further, the web interface allows us to create new files for diagnosis, upload new image files, mint new NFTs, and transfer ownership of NFTs, all of which originate and require the user to access a cryptocurrency wallet such as MetaMask. Once the user signs into the wallet, the wallet provides multilayer security for accessing original content stored within the IPFS and access the application. Within each wallet is access to the NFT for which the current wallet has ownership. The NFT will contain a unique CID for the JSON metadata file, containing further pointers to scanned images, diagnostics from physicians, and prescriptions. Code listing 3 shows how the NFT is minted based on the user input.

Code listing 5 (Multimedia Appendix 2) shows how to access the smart contract on the remote and internet-based machine from the front-end JavaScript of the decentralized application. We use Ethereum events and event listeners within the web3.js implementation standard to invoke the mint functionality of the smart contract. Code listing 5 shows that MintURI is the JavaScript function that captures and returns the token ID once the token is minted. MintURI retrieves the token ID and associates the token ID to the IPFS CID of the JSON file, which contains the metadata (as described in code listing 1).

Regarding privacy of data, this CID can be encrypted by a key pair stored in the wallet by the application when minting to give only the owner access to the URI where the JSON file is stored.

### Transferring Ownership of the Pathological Record

In the code snippet (code listing 6 in Multimedia Appendix 2), we see the URI, the JSON file’s CID being transferred to the MedContract.methods.mint(...). The token is associated with the wallet (Ethereum) address 0x138a93...A8. Overall, once the token is minted, the owner can securely transfer the NFT over the network to another user. This transfers both the owner of the diagnosis file and the URI to the receiver. However, the actual data stored on the IPFS has never moved. The only transfer happening is the token’s ownership (which points to the CID of the JSON file data), which was initially associated with the sender’s wallet address. It will now be accessible only to the receiver. Code listing 6 describes the main functionality and how the user interface sends the code to other users.

The code listing 6 shows how data ownership is transferred from the senders to the receiver. The underlying CID (IPFS) and its contents can now be accessed by the receiver, who then examines the high-resolution image and either contributes by creating a new diagnosis file or creates a new image for minting the NFT.

### Creating an Expiration Date–Driven NFT Access Control

One of the key requirements is for the token to expire as per the expiry date. Different state and local laws dictate how long each hospital can store a patient record and conditions around the storage of the record. For example, in the health care system that the coauthor of this paper works for, the hospital system stores the records (glass slides containing high-resolution microscopic scans and digitized images of these scans) for 3 years, after which they are securely discarded from their storage. However, the owner or patient can choose to retain their copy for as long as they choose.

The approach we chose to replicate this functionality on the blockchain is as follows: The smart contract code supports the burn functionality for the token, which subsequently makes it inaccessible by any of the system’s users. Then, the web3.js application scans each record for the creation date and the owner’s wallet ID and burns those for all those token owners whose wallet IDs do not match the owner’s wallet ID, which is stated in the JSON record. Code listing 7 (Multimedia Appendix 2) displays the burn functionality for the token ID when the token was burned.

Upon execution of the contract within each user’s (eg, physician’s or even pathologist’s) decentralized application, those token ID’s with creation dates exceeding the expiration date would be burned and inaccessible to anyone else on the system. Only the record owner (or patient) would never execute the burn function, preserving original access to the NFT.

### Use Cases Supported by Design and Implementation of the System

**Figure 6:**
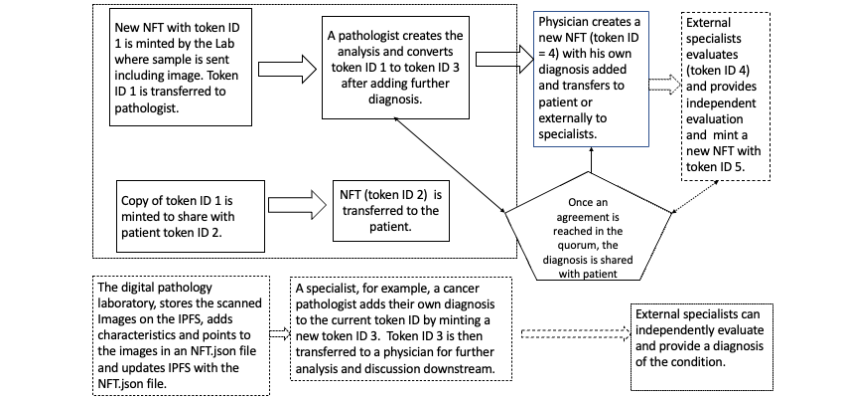
Illustration of a digital pathology workflow with nonfungible tokens applied to scanned images overall. NFT: nonfungible token.

Several use cases are supported by the design and implementation of our system. One such user flow is shown in Figure 6, which directly refers to the aforementioned design requirements.

### Securing Data Storage for Privacy and Data Control

Access control, ownership, and confidentiality of data are three challenges in storing health care–related data on blockchain platforms. Because data reside on the blockchain and either the data or pointer to the data is publicly accessible and available to all users, how can users ensure that their data are accessed only by authorized individuals and that others do not have access to it? Furthermore, how do users ensure an audit trail for the accessed data, as auditability is one of the legal requirements from Health Insurance Portability and Accountability Act or General Data Protection Regulation? Similarly, how are ownership rights of data separated from the right to share the data with health care professionals for specific contexts of diagnosis?

To solve these challenges, we describe and implement the prototype for the security architecture in Table 2 in the context of our digital pathology system on a public blockchain. The security architecture of the digital pathology system is multilayered based on the different access requirements in the system. The principal fundamental functions of the security system include authentication, data integrity, confidentiality, notarization or signature, access control, assurance of availability, and ownership. By authentication, we mean that the data are accessed only by those individuals who handle the data. Data integrity means that the data are never tampered with by anyone in the system. The confidentiality property alludes to the fact that the actual data are not exposed to others, although pointers to the data reside in the blockchain. By notarization, we ensure that every time the data are transmitted or accessed and restored on the system, there is a valid signature of the individual who has accessed the record stored on the system. By access control, we mean that data access is tracked in the system, and only those allowed to access the data through a valid identifier can access the data. Finally, by assurance and availability, we ensure that data are available only to those individuals who have the right to know legally, and the security mechanism is available to all participants in the network (ie, the rules for access control and security are available to all users uniformly). The unique aspect of data record ownership is now added to these properties to the blockchain. The data record ownership ensures that only the primary owner of the data record, that is, the patient, has perennial access to the NFT. In addition, for all those who are not primary owners of the health care record (ie, the nonpatients) to whom the NFT has been transferred, the application would automatically invoke the smart contract’s burn functionality after a preassigned period.

In Table 2, we discuss the different levels of data security and privacy enforcement at three layers, namely, the application layer, smart contract layer, and data storage layer, that is, the IPFS layer for such a system.

**Table 2:** Security layers, property of the security architecture, and implementation.

Layers of the stack	Property of the security architecture	Implementation
Application layer and smart contract layer	Authentication	Users who require access to data are authenticated by their wallet (which contains a hash of the user’s public key and a private key). Each time a message is sent, it is signed and verified using ECDSA^a^. Only those users who can sign in to their wallets can access their corresponding data in the form of the NFT^b^.
IPFS^c^ layer and smart contract layer	Data integrity	Data integrity is managed and maintained by the underlying blockchain and IPFS layers. One of the properties of the blockchain is that the data cannot be manipulated ever. Similarly, the original image or diagnosis file stored in the IPFS cannot be altered. Changing the file will give rise to a new CID^d^, which will need minting a new token with different access controls.
Application layer for encryption and smart contract layer for storing the encrypted CID on the blockchain	Confidentiality of data	Although data stored on the blockchain is public information, the NFT being minted is minted off a JSON file’s CID stored in the IPFS. The application can encrypt and store the encrypted CID on the block in the associated NFT accessible only to the wallet owner. The encryption is done by the application and not by the blockchain or the blockchain’s smart contract. Each time a new token is minted, the resulting metadata file is uploaded onto the IPFS. The CID of the metadata file is encrypted by the application and stored on the blockchain as part of the contract.
Smart contract layer	Notarization	After signing with the owner’s public key, each piece of information is notarized and stored on the blockchain. Each time the NFT is transferred to a new owner, at the application layer, the NFT is signed by the new owner’s public address and later encrypted using the new owner’s keys for storage on the blockchain at the application level, so that only the new owner can access the contents of the NFT.
Application layer and smart contract layer	Access control	Access control for a token is currently maintained by means of a wallet (both public and private keys). However, this access control can be maintained and moderated by the user.
Blockchain and IPFS layer	Availability	This is moderated by the underlying blockchain and IPFS infrastructure that has a 99.99% availability. The only limitation is network availability, which controls the rate at which data can be deposited and pulled from the network.

^a^ECDSA: Elliptic Curve Digital Signature Algorithm.

^b^NFT: nonfungible token.

^c^IPFS: Interplanetary File System.

^d^CID: content identifier.

## Results

**Figure 7:**
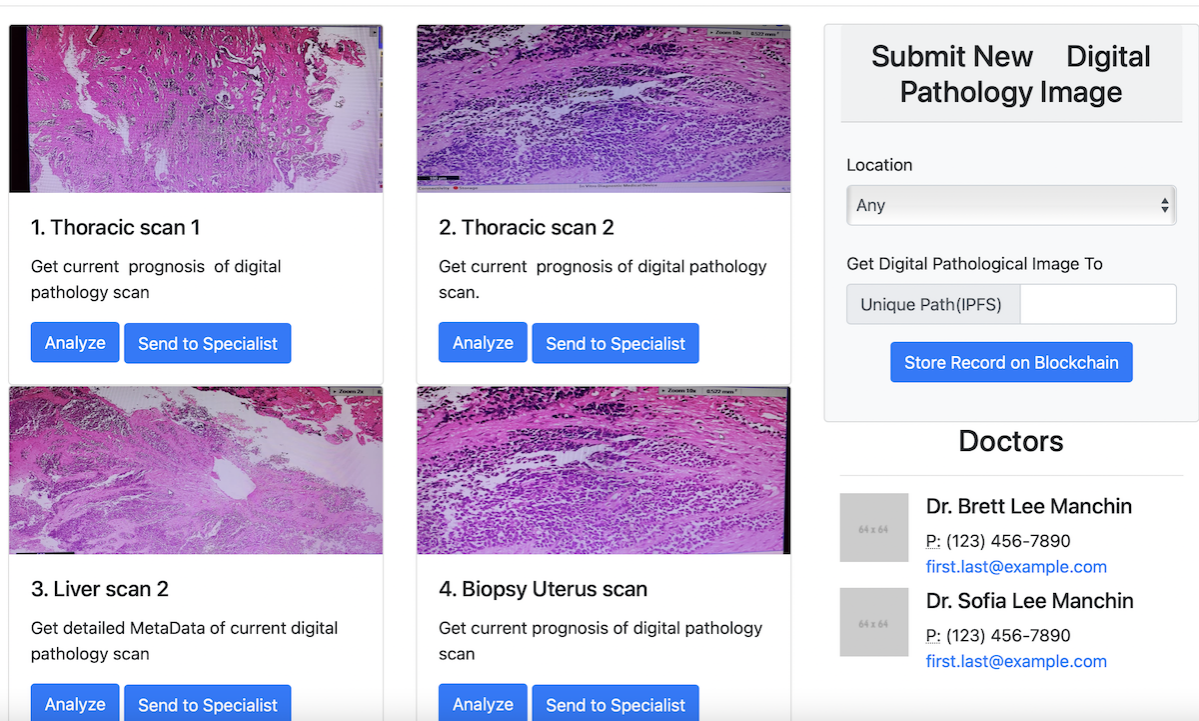
User interface of the digital pathology system that displays 4 specific scans the corresponding physician has access to.

After proof-of-concept implementation, we test and describe how the main objectives of digital pathology solutions are met. Furthermore, using our solution, we evaluate how this solution addresses the challenges associated with storage, transmission, cost-effectiveness, and security concerning digital pathology. Figure 7 shows the screenshots of the prototype digital pathology and the system’s user interface. In addition, these screenshots demonstrate how such a system works and the mode of operation of the digital pathology system.

In Figure 7, we see how these images are accessible to the owner of the data set and now surface at the user interface. The pathologist and other experts who have access to the image through the assignment of the NFT can now click on each image, open the image, and analyze the image through visual inspection or by the assistance of various artificial intelligence algorithms. The respective professional can then save the modified image and diagnosis onto the IPFS, mint a new NFT, and share it with the patient or other specialists in the group. Similarly, access to all such images occurs by signing in to the corresponding wallet of the user. Once signed in, the user has access to the corresponding NFT through a wallet. Such a design that separates the user actions from storage and records each action atop the blockchain creates a valuable chain of record. Table 3 presents the benefits of our design vis-à-vis the original requirements, the implementation details, and the benefits of such a solution.

**Table 3:** Solution requirements and how they are addressed in the implementation.

Requirement	Implementation details	Benefits
Security	There are 2 layers of security for data in such a system. First, the IPFS^a^ that stores images provide a high-security level by encrypting and splitting data into chunks stored on the network. Data are accessible only via a CID^b^, which is a large 256-byte hex code. Next, the JSON metadata file, which addresses the CID that is encrypted within the blockchain, is only accessible by those who use their keys to access the contract address where the data are stored. Refer to Table 2 for the implementation details.	The 2-layer security of smart contract data will only enable the wallet owner to access the record’s contents. IPFS’s CID is impossible to guess randomly.
Privacy of data	Only the user who has access to the corresponding wallet can access the data on the blockchain, and all other users will be blind to the use of data. Refer to Table 2 for an explanation of how data confidentiality and privacy are handled in the design.	This feature of the blockchain provides users (patients), physicians, and others the additional layer of security.
Low-cost, high-fidelity file storage	The IPFS is a public infrastructure secured by nodes running globally. The smart contract code can communicate with the IPFS if contracts prefer pinning (or converting to static storage) the data. An alternate public storage mechanism is FileCoin, which builds an incentive mechanism atop the IPFS to reimburse users for providing high-end storage and availability.	There is no need for compression of data or the manipulation of original data files for long-term storage. Such a storage method enables storage of the original files at high fidelity for further analysis.
High performance	Data and smart contract access is enabled to the high-throughput data storage via the IPFS and metadata file that contains a data pointer (ie, CID of the actual image). We also refer to other decentralized storage systems such as FileCoin and Storj, which are paid alternatives to the IPFS wherein crypto tokens enable the QoS^c^ layer to ensure data availability. As a result, the actual file is never moved on the network, and only access controls to the CID containing JSON files are altered.	Data transfer does not occur on the network, except for the image being scanned and stored on the IPFS. Data access is provided via metadata files that are minted into NFTs. The NFTs, once transferred on the network, transfer the ownership of the underlying token to the receiver. As a result, the only piece of data transferred on the blockchain is the token through a transaction. This improves the performance of any system compared with a networked system infrastructure or a permissioned database structure where entire data files will have to move on the network.
Low transmission cost	Data transmission is accomplished by just transferring the token ownership on the blockchain corresponding to the file. Therefore, the original file is not transmitted over the network; instead, the JSON file’s CID ownership is transferred to a different wallet on the blockchain.	The network transmission of the IPFS record means that only the minted token is transferred in ownership. The details of the token ID are stored and encrypted on the blockchain. Therefore, no actual data are transferring on the blockchain, reducing and improving bandwidth significantly.
Improve data accuracy	Every time the data are modified by the physician or other intermediaries, newer version-controlled files are created on the IPFS.	Higher accuracy of the data helps train artificial intelligence and machine learning models to improve prediction accuracy.
Ownership	Every specific period when NFT^d^ ownership expires for legal reasons, such access is removed automatically through the smart contract’s burn functionality. Only patients who own the token obtain a chance to hold onto the token and access-associated digital images. The rest of the owners will not have access after the expiry (burn of the token).	The ownership of the digital asset is separated from the functionality of the smart contract. Although the NFT can be shared with multiple practitioners, the actual access is determined by the legal policy of the health care system for nonpatient access. Only patients can access their own NFTs after the record’s expiry date, where the NFT access is restricted for all others.

^a^IPFS: Interplanetary File System.

^b^CID: content identifier.

^c^QoS: quality of service.

^d^NFT: nonfungible token.

## Discussion

### Principal Findings

In this study, we introduced several innovations in decentralized digital pathology that help offset the costs of imaging, sharing, and distributing high-resolution slide scans for medical diagnosis. Using private infrastructure mechanisms responsible for the maintenance of nodes and consensus mechanisms and administration of such systems controlled by a few firms would defeat the entire purpose of using digital pathology to improve the speed of diagnosis and the cost of patient care. Although other health care applications have discussed frameworks to store health care data on the blockchain or to use permissioned networks with different types of encryptions, such a design would increase health care costs and system expenses. In addition, with private blockchains or consortium blockchains, the cost for maintaining the infrastructure and governance would centralize the blockchain system.

Although, on private permissioned blockchain, it is feasible to implement such on-chain storage without additional costs for storage, our design uses decentralized public storage as the back end, combined with established NFT standards. At the same time, private or permissioned blockchain architectures could lead to incompatibility among different chains and prevent external sharing of data because private blockchain with on-chain storage operates within network boundaries. Essentially, there would be a need to implement such permission blockchains across the hospital supply chains, including laboratories, partners, verification agencies, and clinics. These costs would prohibit the mass adoption of digital pathology, whose promise is to reduce costs, increase speeds, and use machine-assisted artificial intelligence and machine learning algorithms to communicate with others. In addition, software and hardware upgrades, technology obfuscation, network speeds are left to the blockchain maintainers in the case of private blockchains. As a result, a trade-off between private permission and consortium blockchains without permission would make a design such as the one presented here suitable. The public maintains the infrastructure with a consortium-driven networks and private and public networks.

We relied on a public blockchain infrastructure combined with decentralized storage in our solution, which we believe will drastically reduce the costs for storage transmission and provide a privacy-enabled and secure system for public health care. At the same time, the participants can also contribute to such infrastructure by offering nodes on which they could pin their files for the IPFS. Alternatively, an inexpensive decentralized storage system such as Storj could be used, providing permissioned access to ample storage at a very affordable cost. The only piece of data that the blockchain stores is the encrypted CID for the IPFS metadata file. Although the IPFS metadata file is addressable and accessed outside the blockchain by users whose wallets have the authority to access the contents (image scans, diagnosis, treatment pathways, and other associated metadata), our security architecture prevents the public or other users from accessing the contents, thus providing security.

We reduced the cost of storage of high-resolution images by proposing the use of the IPFS, a secure and distributed peer-to-peer network with high availability. We used cryptocurrency wallets and smart contracts to create NFTs using the widely accepted ERC-721 standards. This design precludes the need to add a separate security layer for data storage, transmission, and access. Although we separated the storage of the actual image from its metadata, the transmission of the image entails changing the permission for accessing the metadata file through encryption with the receiver’s keys. Our design approach does not require moving the entire high-resolution image to a different address on a network, which would likely consume much more network bandwidth and is inefficient, although this can also be accomplished.

### Conclusions

Overall, the data transmission, data storage, and use of the ERC-721(NFT) standard for pathology-based image storage will improve the prospects for mass adoption of digital pathology. Such an architecture can significantly reduce the cost of the infrastructure required for digital pathology and, over time, improve the speed for diagnosis and affordable medical care.

## Appendix

Multimedia Appendix 1 Questions answered by pathologist physician coauthor (SS) that discusses challenges with digital pathology.

Multimedia Appendix 2 Source code listings.

## Abbreviations

CID: content identifier
DSRP: design science research process
ERC: Ethereum Request for Comments
IPFS: Interplanetary File System
NFT: nonfungible token
RQ: research question

## References

[1] Madabhushi A, Lee G (2016). Image analysis and machine learning in digital pathology: challenges and opportunities. Med Image Anal.

[2] Pantanowitz L (2010). Digital images and the future of digital pathology. J Pathol Inform.

[3] Schmitt M, Maron RC, Hekler A, Stenzinger A, Hauschild A, Weichenthal M, Tiemann M, Krahl D, Kutzner H, Utikal JS, Haferkamp S, Kather JN, Klauschen F, Krieghoff-Henning E, Fröhling S, von Kalle C, Brinker TJ (2021). Hidden variables in deep learning digital pathology and their potential to cause batch effects: prediction model study. J Med Internet Res.

[4] Tizhoosh H, Pantanowitz L (2018). Artificial intelligence and digital pathology: challenges and opportunities. J Pathol Inform.

[5] Jahn SW, Plass M, Moinfar F (2020). Digital pathology: advantages, limitations and emerging perspectives. J Clin Med.

[6] Hartman D, Pantanowitz L, McHugh J, Piccoli A, OLeary M, Lauro G (2017). Enterprise implementation of digital pathology: feasibility, challenges, and opportunities. J Digit Imaging.

[7] Romero Lauro G, Cable W, Lesniak A, Tseytlin E, McHugh J, Parwani A, Pantanowitz L (2013). Digital pathology consultations-a new era in digital imaging, challenges and practical applications. J Digit Imaging.

[8] Zuo Z, Lan X, Deng L, Yao S, Wang X (2015). An improved medical image compression technique with lossless region of interest. Optik.

[9] Kempfert A, Reed B (2011). Health care reform in the United States: HITECH Act and HIPAA privacy, security, and enforcement issues. FDCC Quarterly.

[10] Nahra KJ (2008). HIPAA security enforcement is here. IEEE Secur Privacy Mag.

[11] Janowczyk A, Madabhushi A (2016). Deep learning for digital pathology image analysis: a comprehensive tutorial with selected use cases. J Pathol Inform.

[12] Domingue J, Third A, Ramachandran M (2019). The FAIR TRADE framework for assessing decentralised data solutions. Proceedings of the Companion Proceedings of the 2019 World Wide Web Conference.

[13] Subramanian H (2017). Decentralized blockchain-based electronic marketplaces. Commun ACM.

[14] Costello SS, Johnston DJ, Dervan PA, O'Shea DG (2003). Development and evaluation of the virtual pathology slide: a new tool in telepathology. J Med Internet Res.

[15] Park YR, Lee E, Na W, Park S, Lee Y, Lee J (2019). Is blockchain technology suitable for managing personal health records? Mixed-methods study to test feasibility. J Med Internet Res.

[16] Maslove DM, Klein J, Brohman K, Martin P (2018). Using blockchain technology to manage clinical trials data: a proof-of-concept study. JMIR Med Inform.

[17] Ichikawa D, Kashiyama M, Ueno T (2017). Tamper-resistant mobile health using blockchain technology. JMIR Mhealth Uhealth.

[18] Khurshid A (2020). Applying blockchain technology to address the crisis of trust during the COVID-19 pandemic. JMIR Med Inform.

[19] Sylim P, Liu F, Marcelo A, Fontelo P (2018). Blockchain technology for detecting falsified and substandard drugs in distribution: pharmaceutical supply chain intervention. JMIR Res Protoc.

[20] Subramanian H, Liu R (2019). Blockchain and smart contracts. J Database Manag.

[21] What is ERC-721?. ERC-721.

[22] Sengupta A, Subramanian H (2022). User control of personal mHealth data using a mobile blockchain app: design science perspective. JMIR Mhealth Uhealth.

[23] Peffers K, Tuunanen T, Rothenberger MA, Chatterjee S (2014). A design science research methodology for information systems research. J Manag Inf Syst.

[24] (2009). Model-view-controller pattern. Learn Objective-C for Java Developers.

[25] Kumar R, Tripathi R (2020). Blockchain-based framework for data storage in peer-to-peer scheme using interplanetary file system. Handbook of Research on Blockchain Technology.

