# Supplementary material for: Improving Diagnosis Through Digital Pathology: Proof-of-Concept Implementation Using Smart Contracts and Decentralized File Storage
Source: J Med Internet Res. 2022 Mar 28;24(3):e34207. doi: 10.2196/34207 (PMC9002606; doi:10.2196/34207)
Supplement: Multimedia Appendix 1 [file jmir_v24i3e34207_app1.docx]

**Multimedia Appendix 1. Questions answered by pathologist physician coauthor (SS) that discusses challenges with digital pathology.**

- How frequently do you use Digital Pathology?
- Compared to traditional Pathology, how does Digital Pathology help you and your staff to diagnose better?
- What are some of the challenges of Digital Pathology?
- What are some of the benefits of Digital pathology?
- What are some of the cost barriers to Digital pathology for mass adoption – especially in a country like India?
- Do you prefer glass slides or computerized scans of slides as shown in digital Pathology?
- How long do you think your hospital system keeps these records?
