# Supplementary material for: Improving Diagnosis Through Digital Pathology: Proof-of-Concept Implementation Using Smart Contracts and Decentralized File Storage
Source: J Med Internet Res. 2022 Mar 28;24(3):e34207. doi: 10.2196/34207 (PMC9002606; doi:10.2196/34207)
Supplement: Multimedia Appendix 2 [file jmir_v24i3e34207_app2.docx]

**Multimedia Appendix 2.** Source code listings.

Code Listing 1. JSON metadata file that stores the parameters of the digital pathology scan.

*{*

*"name": "NFT for Thoracic Scan 1",*

*"description": "This image shows the thoracic scan of the patient with sections marked to highlight tissue decomposition that needs further investigation.",*

*"image file":*

*“Resolution” : 10000000 dpi*

*"device": "3DHISTECH Panoramic"*

*"Clinic": "Monta vista pharmaceuticals and diagnostics ltd. Ohio, USA"*

*"Physician ID": 198765482*

*"Owner": Patient Name*

*"Owner wallet ID": Patient ID*

*}*

Code Listing 2. Smart contract functions for creating the token and transfer of the data.

| Contract NFTToken { |
| --- |
| event Mint(address indexed _to, uint256 indexed _tokenId, bytes32 _ipfsHash); |
| event Transfer(address indexed _from, address indexed _to, uint256 indexed _tokenId); |
| event SAFETransferFrom(address _from, address _to, uint256 _tokenId); |
| event MintUri(address indexed to, uint256 indexed tokenId, string indexed uri); |
| uint256 tokenCounter = 1; |
| mapping(uint256 => address) internal idToOwner;  } |
| Code Listing 3. Safe transfer function for transferring the token from a particular address to another address. |
| Function safeTransferFrom(address _from, address _to, uint256 _tokenId, bytes32 _hash) public payable returns(bytes32 hash){ |
| assert(msg.sender==idToOwner[_tokenId]); |
| assert(_from == idToOwner[_tokenId]); |
| idToOwner[_tokenId] = _to; |
| emit SAFETransferFrom(msg.sender,_to, _tokenId); |
| return _hash; |
| } |

Code Listing 4. Source code for minting the NFT using the CID from the user input.

$("#button").click(function() {

$("loader").show();

console.log($("#token").val());

MedContract.methods.mint($(“#address”).val(),$("#token").val()).

Send({from:web3.eth.defaultAccount},function(error,transactionHash){

if(!error){

console.log(transactionHash);

} else {

console.log(error);

}

});

});

Code Listing 5. Source code for minting the NFT token using the CID from the user input.

*MedContract.events.MintUri({*

*fromBlock:"latest"*

*}, function(error, event){*

*console.log("return new token here ");*

*if (!error)*

*{*

*$("#loader").hide();*

*$("#instructor").html("address" + event.returnValues['0']);*

*$("#MedRecord").html("token ID" + event.returnValues['1']);*

*console.log("token ID is" + event.returnValues);*

*} else {*

*$("#loader").hide();*

*console.log("token ID is not returned" + event);*

*}*

*});*

Code Listing 6. Source code depicting how the NFT is transferred from one user to another user. Please note that the addresses in the method safeTransferFrom, that is, 0x13..., 0xb4.... and so on, are useful parameters overall.

*$("#transferbutton").click(function() {*

*$("loader").show();*

*MedContract.methods.safeTransferFrom('0x138a932E779D5575355261F64D8544d8003bC4A8','0xb43CE2E96E0F398956DD57ceD0033bf2eB9BC170',29,'0x000000456789').*

*Send({from:web3.eth.defaultAccount,function(error,transactionHash){*

*if(!error){*

*console.log(transactionHash);*

*} else {*

*console.log(error);*

*}*

*});*

*});*

*MedContract.events.SAFETransferFrom({*

*fromBlock:"latest"*

*}, function (error, event){*

*console.log ("Comes to event here");*

*if (!error)*

*{*

*$("#loader"). Hide();*

*$("#instructor").html ("address" + event.returnValues['0']);*

*$("#MedRecord").html ("token ID" + event.returnValues['1']);*

*console.log ("tries it here 1" + event.returnValues);*

*} else {*

*$("#loader"). Hide();*

*console.log ("tries it here 2" + event);*

*}*

*});*
